# Semi-Supervised Vertebra Segmentation and Identification in CT Images

**DOI:** 10.3390/tomography12030033

**Published:** 2026-03-03

**Authors:** You Fu, Jiasen Feng, Hanlin Cheng

**Affiliations:** 1 School of Information Technology, Murdoch University, Murdoch 6150, Australia; 33729313@murdoch.edu.au; 2 School of Automation, University of Science and Technology Beijing, Beijing 100083, China; 41452005@ustb.edu.cn; 3 School of Biological Sciences and Medical Engineering, Southeast University, Nanjing 210096, China

**Keywords:** CT, vertebra segmentation, vertebra identification, semi-supervised learning, deep learning

## Abstract

**Background/Objectives**: Automatic segmentation and identification of vertebrae in spinal CT are essential for assisting diagnosis of spinal disorders and for preoperative planning. The task is challenging due to the high structural similarity between adjacent vertebrae and the morphological variability of vertebrae. Most existing methods rely on fully supervised deep learning and, constrained by limited annotations, struggle to remain robust in complex scenarios. **Methods**: We propose a semi-supervised approach built on a dual-branch 3D U-Net. Mamba modules are inserted between the encoder and decoder to model long-range dependencies along the cranio–caudal axis. The identification branch employs a 3D convolutional block attention module (3D-CBAM) to enhance class discriminability. A unified semi-supervised objective is formulated via teacher–student consistency: for each unlabeled sample, weakly and strongly augmented views are generated, and cross-branch consistency is enforced, together with confidence-based filtering and class-frequency reweighting. In addition, a connected-component analysis is used to enforce anatomically plausible sequential continuity of vertebral indices in the outputs. **Results**: Experiments on VerSe 2019 and 2020 show that, on the public VerSe 2019 test set (with VerSe 2020 scans used as unlabeled training data), the supervised baseline achieved a Dice score of 89.8% and an identification accuracy of 92.3%. Incorporating unlabeled data improved performance to 91.6% Dice and 97.5% identification accuracy (relative gains of +1.8 and +5.2 percentage points). Compared with competing methods, the proposed semi-supervised model attains higher or comparable segmentation accuracy and the highest identification accuracy. **Conclusions**: Without additional annotation cost, the proposed method markedly improves the overall performance of vertebra segmentation and identification, offering more robust automated support for clinical workflows.

## Simple Summary

Accurate vertebra segmentation and level identification on spine CT can support diagnosis and surgical planning, but manual labeling is time-consuming, and automated methods may fail when scans cover only part of the spine. We propose a semi-supervised deep-learning method that learns from a small set of labeled scans and additional unlabeled scans to improve robustness. On public benchmarks, it increased both segmentation quality and vertebra identification accuracy compared with supervised training alone. This approach may reduce radiologists’ workload, improve consistency in reporting, and facilitate large-scale studies and future clinical applications.

## 1. Introduction

Spine-related diseases are highly prevalent in clinical practice, and accurately obtaining the anatomical morphology and index (ID) of each vertebra is crucial for diagnosis and surgical planning [1,2]. CT is widely used owing to its high spatial resolution for osseous structures. However, manual vertebra-by-vertebra annotation is time-consuming and subject to inter-observer variability, motivating research into automated vertebral segmentation and identification.

Deep learning [3], especially encoder–decoder models, such as U-Net, has advanced medical image segmentation [4], but performance is often limited by scarce annotations. The release of the VerSe benchmark datasets has enabled standardized evaluation and methodological iteration [5]. Multi-stage pipelines built on VerSe (e.g., first localizing/identifying vertebrae, then segmenting each vertebra) have shown promising results. However, treating “segmentation” and “identification” as separate steps limits cross-task information sharing [6,7], and global numbering shifts can still occur under limited fields of view or in complex cases.

Earlier studies commonly decoupled segmentation and identification. Along the “segmentation-only” line, traditional approaches (statistical shape models [8,9,10], thresholding/active contours, etc.) and deep networks (FCN [4], U-Net [4], V-Net [11], and iterative frameworks [10,12,13]) continuously improved the voxel-level accuracy. Along the “identification-only” line, typical strategies attached classification/ranking heads to already segmented regions or high-level features [8] or iteratively inferred vertebral indices along the spinal axis [13,14,15]. While foundational, these separate pipelines allow segmentation errors to propagate to identification and make it difficult to use identification priors to enforce segmentation coherence.

More recent work has shifted toward performing vertebral segmentation and identification within a single model, leveraging shared representations for mutual benefit. Representative studies such as SIIL output voxel-wise masks and vertebral IDs in an end-to-end fashion, reducing error propagation arising from segmentation–identification inconsistencies [16]. Other graph-optimization-based iterative paradigms unify localization, segmentation, and identification within a single iterative solver using anatomical consistency constraints to automatically correct labeling errors and highlight the advantages of “joint modeling + structural priors” [15]. In parallel, discriminative representation learning for identification (e.g., contrastive learning and uncertainty modeling) has been integrated into joint frameworks to improve separability between adjacent vertebrae and to stabilize the final ID sequence [17]. Overall, integrated joint segmentation–identification modeling appears to be a more suitable path to enhanced robustness.

Moreover, the high cost of medical image annotation limits large-scale supervised training. Semi-supervised learning (SSL) mitigates the scarcity of labels by incorporating unlabeled data into training [18]. Typical strategies include Mean Teacher consistency [18], uncertainty-aware pseudo-label filtering/weighting [19,20], transformation-consistency self-ensembling [21], dual-task consistency [22], and more general approaches, such as UniMatch v2 [23]. In general, SSL has proven effective for alleviating annotation scarcity in medical imaging; the key lies in designing task-appropriate consistency constraints and pseudo-label strategies. These strategies are compatible with joint modeling: within a shared-encoder multi-task framework, consistency regularization and high-confidence pseudo-labels can jointly regularize both the segmentation and identification branches, improving overall consistency and data efficiency.

Despite recent progress, vertebra segmentation and identification in routine CT still face three practical failure modes: (i) partial field-of-view (FOV) scans may miss upper or lower vertebrae, inducing a global index shift; and (ii) the high structural similarity between adjacent vertebrae often causes swaps, merges, or fragmentation. These observations motivate a unified framework that jointly reasons about 3D anatomy and sequential continuity while reducing reliance on dense annotations.

Different from prior joint segmentation–identification approaches that primarily rely on anatomical priors or deterministic indexing, we introduce a semi-supervised teacher–student objective tailored to the joint setting: segmentation and identification are regularized simultaneously via complementary consistency terms. This design not only improves segmentation under limited labels but also mitigates identification failures, such as global index shift and adjacent-vertebra confusion.

To address the above challenges, we propose an end-to-end multi-task framework that performs vertebra segmentation and identification within a single model, leveraging a shared encoder for cross-task synergy and incorporating semi-supervised learning to reduce reliance on manual annotations. Our main contributions are as follows:

1. Dual-branch 3D U-Net with Mamba and 3D convolutional block attention module (CBAM). We design a dual-branch 3D U-Net that integrates Mamba modules [24] and 3D-CBAM [25] to model the sequential dependencies of vertebrae and to accomplish segmentation and identification within a unified architecture. A shared encoder serves as the core feature extractor: the encoder representation feeds (i) a segmentation branch to produce voxel-wise vertebral masks and (ii) an identification branch to predict anatomical indices from global features. Through shared representations and parallel optimization, the two branches mutually reinforce each other, enabling integrated, synergistic vertebra segmentation–identification.
2. Unified semi-supervised objective for joint segmentation–identification. Building on the UniMatch v2 semi-supervised semantic segmentation framework [23], we formulate a unified SSL objective tailored to our two-branch setting. For each unlabeled CT volume, we generate weakly and strongly augmented views and enforce teacher–student consistency on both branches [18,23]. In the segmentation branch, high-confidence pseudo-masks are obtained by thresholding the teacher’s foreground probabilities, and binary cross-entropy is computed only on voxels that pass confidence filtering. In the identification branch, temperature-calibrated teacher heatmaps provide pseudo-classes, and a weighted cross-entropy is applied at high-confidence locations. Combined with uncertainty-guided filtering [19,20], class-frequency reweighting, and a linear ramp-up of the consistency weight, this design markedly improves the utilization of unlabeled data and enhances cross-task coherence and overall performance. Notably, unlike UniMatch v2 (which addresses only single-task segmentation), our approach extends consistency training to a dual-task setting with branch-specific pseudo-labeling strategies, representing a novel application of SSL to joint segmentation–identification.

The remainder of this paper is structured as follows: Section 2 presents materials and methods, including the overall framework, datasets, network architecture, loss functions, data augmentation, implementation details, and experimental design. Section 3 reports experimental results. Section 4 provides the discussion. Section 5 concludes the study and outlines future work.

## 2. Materials and Methods

### 2.1. Overall Framework

**Figure 1:**
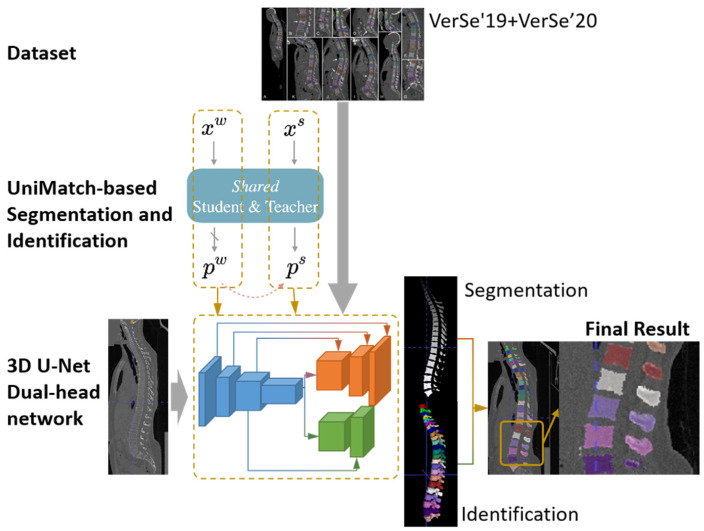
Overview of the proposed method.

We propose a unified end-to-end framework that performs voxel-level segmentation and instance-level identification of vertebrae within a single 3D convolutional neural network. The overall pipeline is illustrated in Figure 1. Given a spinal CT sub-volume as the input, a deep 3D convolutional encoder first extracts multi-scale features, which are then fed in parallel into two decoder branches: (i) a segmentation branch that progressively upsamples features to reconstruct a vertebral probability mask with the same spatial dimensions as the input and (ii) an identification branch that processes high-level global features from the encoder to predict the anatomical ID of each vertebra within the sub-volume. The final output combines the vertebral masks and their corresponding predicted IDs to form complete labeled results. Note that the dual-branch 3D U-Net is trained under our semi-supervised learning framework, in which both the teacher and student models share the same dual-branch 3D U-Net architecture.

### 2.2. Dataset

We conduct experiments on VerSe [5], a publicly available benchmark dataset for vertebral segmentation and identification, comprising VerSe 2019 and VerSe 2020. VerSe 2019 contains 160 CT scans (80 training, 40 validation, and 40 test), with 1725 annotated vertebrae spanning C1–L5. VerSe 2020 expands the scale to 319 CT scans (113 training, 103 validation, and 103 test), with 4141 annotated vertebrae from C1 to L5. Details are summarized in Table 1.

The native CT volumes have isotropic or near-isotropic voxel spacing of approximately 0.5–1.5 mm. To ensure consistent spatial scale and mitigate scanner-dependent resolution differences, all volumes are resampled to 1.0 × 1.0 × 1.0 mm (using trilinear interpolation). A unified normalization pipeline is then applied. Because a full spinal CT is large (on average ≈ 512 × 512 × 300 voxels) and cannot be processed by a 3D network in a single pass due to GPU memory limits, we extract sub-volumes (patches) via random/sliding-window cropping along the axial direction—random during training and sliding at inference—followed by isotropic scaling. Symmetric padding is applied along the depth dimension as needed to match a fixed input size of 128 × 64 × 64 voxels. Finally, intensities are clipped to [−500, 1500] Hounsfield units (HUs) and linearly normalized to [0, 1].

### 2.3. Network Architecture

As shown in Figure 1, we use a dual-head 3D U-Net with a shared encoder and two task-specific decoders and insert a Mamba module [24] at the bottleneck to model long-range cranio–caudal dependencies.

The encoder has four stages. Each stage contains two 3D convolutions followed by instance normalization and LeakyReLU and applies ×2 downsampling to expand the receptive field while preserving multi-scale skip features. At the bottleneck, Mamba aggregates distant context without the quadratic cost of self-attention. Its linear-time complexity (in sequence length) and lower memory footprint make it suitable for 3D volumes, where capturing cranio–caudal continuity is critical for cross-vertebra consistency and robust indexing, especially under partial FOV/truncated scans that can induce global ID shifts.

We adopt separate decoders to reduce negative transfer and allow task-specific optimization. The segmentation decoder performs three successive upsampling steps with skip-feature fusion to output a voxel-wise binary mask at full resolution. Deep-supervision heads at 1/2 and 1/4 resolutions improve the gradient flow, stabilize small-batch 3D training, and enhance the boundary details.

The identification decoder uses a lighter upsampling path and outputs, at 1/4 resolution, multi-channel confidence/heatmaps for vertebral IDs. Predicting at 1/4 resolution balances localization granularity and compute/memory cost while retaining sufficient spatial detail for centroid-based assignments. For VerSe-style reporting, we follow the C1–L5 protocol (24 classes) and explicitly state any label mapping; we optionally support extended labels (e.g., C1–S1 plus background) to handle transitional anatomy while keeping the reporting protocol consistent.

To improve class separability, we insert 3D-CBAM [25] (channel attention followed by spatial attention) only in the identification decoder (upsampling and output stages) to suppress redundant channels and irrelevant spatial responses. We keep the segmentation decoder purely convolutional to avoid degrading precise boundary modeling.

### 2.4. Loss Functions

#### 2.4.1. Overall Objective

The total loss comprises a supervised term and a self-supervised (consistency) term with a linearly ramped weight, as in Equation (1). The ramp-up policy is as follows: the consistency weight is zero for the first several epochs (supervised-only training), then increases from a specified epoch and reaches a stable level by mid-training, i.e., it grows linearly from 0 to 1 to fully leverage unlabeled data thereafter.

(1) $$L_{total}\left(t\right)=L_{sup}+\lambda \left(t\right)L_{cons}$$

Here, $L_{total}\left(t\right)$ is the total loss at epoch t; $L_{sup}$ is the supervised term; $L_{cons}$ is the self-supervised consistency term; and $\lambda \left(t\right)$ is the epoch-dependent weight (Equation (2)):

(2) $$\lambda \left(t\right)=\left\{\begin{array}{c} \;\;0,\;\;t<E_{cons}\\ \min\left(1,\;\frac{t-E_{cons}}{E_{ramp}-E_{cons}}\right),\;\;t\geq E_{cons} \end{array}\right.$$

where $E_{cons}$ is the epoch at which the consistency term is activated, and $E_{ramp}$ denotes half of the total number of training epochs. In our implementation, we use a supervised-only warm-up of the first 40 epochs ($E_{cons}$ = 40) and linearly ramp the consistency weight for 40 epochs ($E_{ramp}$ = 80).

Notation and symbols used in Equations (1)–(11) are summarized in Table A1 for clarity.

#### 2.4.2. Supervised Term

The supervised loss $L_{sup}$ is the weighted sum of the segmentation and identification losses (Equation (3)), where the identification weight $\alpha_{cls}$ strengthens the learning of vertebral indices:

(3) $$L_{sup}=L_{seg}+\alpha_{cls}\left(L_{cls}^{KL}+L_{cls}^{inst}\right)$$

For the segmentation branch, we adopt multi-scale deep supervision: at full, half, and quarter resolutions, we compute an equal-weighted sum of cross-entropy and Dice loss, then aggregate them with scale weights (larger for higher resolutions), as shown in Equation (4):

(4) $$L_{seg}=\sum_{s\in S}w_{s}\left(\alpha_{seg}Dice_{s}+\left(1-\alpha_{seg}\right)CE_{s}\right)$$

At scale s, the Dice term $Dice_{s}$ and cross-entropy term $CE_{s}$ are as follows:

(5) $$Dice_{s}=1-\frac{2\sum_{x\in \Omega_{s}}p_{s}\left(x\right)g_{s}\left(x\right)+\varepsilon }{\sum_{x\in \Omega_{s}}p_{s}\left(x\right)+\sum_{x\in \Omega_{s}}g_{s}\left(x\right)+\varepsilon }$$

(6) $$CE_{s}=-\frac{1}{\left|\Omega_{s}\right|}\sum_{x\in \Omega_{s}}\left[g_{s}\left(x\right)\ln\left(p_{s}\left(x\right)\right)+\left(1-g_{s}\left(x\right)\right)\ln\left(1-p_{s}\left(x\right)\right)\right]$$

Notation: $\Omega_{s}$ is the voxel grid at scale s; x is the voxel; $g_{s}\left(x\right)\in \left\{0,1\right\}$ is the ground-truth label (foreground = 1, background = 0); $p_{s}\left(x\right)\in [0,1]$ is the predicted foreground probability; $w_{s}$ is the scale weight with $\sum_{s\in S}w_{s}=1$; $\alpha_{seg}$ is the internal weighting factor of the segmentation loss; and $\varepsilon =10^{-6}$ avoids division by zero.

The identification branch combines two complementary terms—distribution alignment $L_{cls}^{KL}$ and instance consistency $L_{cls}^{inst}$.

1. Distribution alignment $L_{cls}^{KL}$. Kullback–Leibler (KL) divergence between the target Gaussian heatmap and the predicted spatial distribution (Equation (7)):
  (7) $$L_{cls}^{KL}=KL(H||P)$$
2. Instance consistency $L_{cls}^{inst}$. At low resolution, we separate the foreground into per-vertebra instances and compute the mean Dice to penalize merging/fragmentation (Equation (8)):
  (8) $$L_{cls}^{inst}=avg_{m}Dice\left(I_{m},\;{Î}_{m}\right)$$

Here, $I_{m}$ and ${Î}_{m}$ denote the ground-truth and predicted per-vertebra instance maps, respectively. H is the normalized Gaussian heatmap target (from ground-truth centroids, or high-confidence teacher centroids for unlabeled data; ∑H=1). P is the softmax-normalized spatial distribution from the identification logits. For labeled samples, H is from ground truth; for unlabeled samples, H is from the teacher. The two terms are dynamically reweighted.

Gaussian heatmap parameters. After resampling to the fixed training spacing, each centroid is rendered as a 3D Gaussian with σ=2.0 voxels; the heatmap is normalized to sum to 1. We use the same σ for all vertebra levels (no level-specific tuning).

#### 2.4.3. Self-Supervised Term

The total consistency loss $L_{cons}$ is the weighted sum of the segmentation consistency term $L_{cons}^{seg}$ and the identification consistency term $L_{cons}^{cls}$ (Equation (9)). We adopt teacher–student consistency: the teacher generates pseudo-labels on a weakly augmented view, while the student receives the corresponding strongly augmented view and is encouraged to match the teacher.

(9) $$L_{cons}=L_{cons}^{seg}+L_{cons}^{cls}$$

1. Segmentation consistency $L_{cons}^{seg}$. We threshold teacher probabilities to form a pseudo-mask and compute BCE on student logits only over high-confidence voxels (Equation (10)):
  (10) $$L_{cons}^{seg}={CE}_{x\in \Omega_{seg}}\left({ŷ}_{x}^{T},\;\sigma \left(z_{x}^{S}\right)\right)$$
2. Identification consistency $L_{cons}^{cls}$. The t temperature-calibrated teacher heatmaps yield pseudo-classes (argmax); we compute CE at high-confidence locations with a ramped threshold, applying class-frequency weights and excluding the background (Equation (11)):
  (11) $$L_{cons}^{cls}=\sum_{k=1}^{K}w_{k}{CE}_{x\in \Omega_{cls}^{k}}\left(y_{x}^{T},\;y_{x}^{S}\right)$$
3. Confidence-threshold schedules. For identification, we apply softmax with temperature T=0.5 to teacher heatmaps and keep pseudo-labels only at locations whose maximum confidence exceeds $\tau_{cls}$. In our implementation, $\tau_{cls}$ is ramped from 0.30 to 0.55 when $t\geq E_{cons}$(Equation (12)). For segmentation, consistency is computed only on confident voxels when $p_{seg}>\tau_{seg}$, with $\tau_{seg}$ ramped from 0.50 to 0.90 (Equation (13)).
  (12) $$\tau_{cls}\left(t\right)=0.3+0.25\times min\left(1,\;\frac{\left(t-E_{cons}\right)}{\left(E_{ramp}-E_{cons}\right)}\right),\;for\;t\geq E_{cons}$$
  (13) $$\tau_{seg}\left(t\right)=min\left(0.90,\;0.50+0.40\times min\left(1,\;1,\;\frac{\left(t-E_{cons}\right)}{\left(E_{ramp}-E_{cons}\right)}\right)\right),\;\;for\;p_{seg}>\tau_{seg}$$
4. EMA teacher update. After each student optimization step, the EMA teacher is updated at every iteration by $\theta_{ema}\leftarrow \alpha \cdot \theta_{ema}+(1-\alpha )\cdot \theta_{student}$ with α=0.99. At the end of warm-up, the EMA teacher is initialized from the current student weights before enabling consistency training.

Notation: $\Omega_{seg}$ is the set of high-confidence voxels used for segmentation consistency; *x* is the voxel index; ${ŷ}_{x}^{T}\in \{0,1\}$ is the teacher’s binary pseudo-label at voxel x (obtained by thresholding teacher foreground probabilities); $\Omega_{cls}^{k}$ is the set of high-confidence voxels for class kkk used for identification consistency; $y_{x}^{T}\in {\left\{0,1\right\}}^{K}$ is the teacher’s one-hot pseudo-class label at x (K vertebral classes, e.g., C1–L5); $y_{x}^{T}\in {\left[0,1\right]}^{K}$ is the student’s class-probability vector at x with $\sum_{k=1}^{K}y_{x,k}^{S}=1$; $\sigma \left(z_{x}^{S}\right)$ is the sigmoid-normalized student segmentation probability from the logit $z_{x}^{S}$; $w_{k}$ is the class-adaptive weight based on training-set frequency with $\sum_{k=1}^{K}w_{k}=K$.

### 2.5. Data Augmentation

In our semi-supervised framework, we use teacher–student consistency with synchronized views for each unlabeled volume: weak $x_{u}^{w}$ for the teacher and strong $x_{u}^{s}$ for the student.

Weak augmentation (geometry only). To simulate pose variability while maintaining topology, we apply random flips (X/Y with probability 0.5, Z with probability 0.25) and an in-plane XY rotation with an angle uniformly sampled from $\left[-22.5^{{}^{\circ}},+22.5^{{}^{\circ}}\right]$ (linear interpolation).

Strong augmentation (photometric/noise only). The strong view inherits the weak geometry (no extra geometric distortion) and applies stochastically sampled intensity/noise operations (RandAugment-style [26]) with independently sampled magnitudes. The pool includes intensity scaling/shifting, gamma correction, Gaussian/salt-and-pepper/Poisson/speckle noise, Gaussian blur, median filtering, unsharp masking, and slice-wise CLAHE. Operations are applied only to image intensities to avoid label contamination.

For labeled samples, we apply the same geometry to volume and mask (trilinear vs. nearest-neighbor interpolation). We apply the full strong photometric/noise pipeline to the volume with *p* = 0.5; otherwise, we optionally apply mild intensity scaling in $\left[0.95,\;1.05\right]$ with a probability of 0.5. Masks are never modified by photometric/noise operations. See Table A2 for parameter ranges.

### 2.6. Implementation Details

We implement the model in PyTorch 2.7.0 and train for 100 epochs on a single NVIDIA RTX 3090 Ti GPU (Santa Clara, CA, USA) (random seed 42). All FSL and SSL variants share identical preprocessing, patch extraction, and optimization settings to ensure fair comparison. We use SGD with momentum (initial learning rate of 0.01, momentum of 0.9). The training schedule includes a supervised-only warm-up for the first 40 epochs; the consistency weight is then linearly ramped from 0 to 1 over the next 40 epochs (epochs 41–80) and kept fixed thereafter. In the same schedule, the pseudo-label thresholds are increased linearly ($\tau_{cls}$: 0.30 to 0.55; $\tau_{seg}$τ_seg: 0.50 to 0.90).

At inference, the segmentation output is thresholded at a probability of 0.5 to obtain a foreground mask. For identification, the predicted 1/4-resolution class-wise heatmaps are upsampled to the segmentation resolution; per-class peak locations are treated as predicted centroids, and each foreground voxel is assigned to the nearest centroid in Euclidean distance to produce a voxel-wise vertebra ID map. We then apply 3D connected-component labeling on the segmentation mask to extract candidate vertebral instances and remove small, isolated fragments. For each connected instance, we compute its geometric centroid and sort instances along the cranio–caudal axis. Predicted IDs are matched one-to-one with the sorted instances, after which a sequence-continuity prior is enforced to ensure anatomically plausible numbering: for duplicate IDs, we retain the instance with higher confidence and reassign the others according to the spatial order; for missing or skipped IDs, we shift or fill labels from superior to inferior to conform to the typical anatomical sequence. This post-processing, grounded in anatomical continuity, yields a one-to-one, superior-to-inferior consistent vertebral index for each connected instance, improving the coherence and utility of the joint segmentation–identification output.

### 2.7. Experiments and Evaluation

#### 2.7.1. Fully Supervised vs. Semi-Supervised Settings

To systematically assess performance, we establish two training protocols on VerSe 2019 and VerSe 2020: fully supervised learning (FSL) and SSL. Unless otherwise specified, we adopt the official VerSe 2019 validation/test splits for model selection and final reporting to ensure consistent evaluation.

1. Data pools. Labeled training pool: VerSe 2019 training split (80 cases) with voxel-wise masks and vertebral IDs. Unlabeled training pool: VerSe 2020 training split (113 cases); labels are not used, and these cases are treated as unlabeled inputs in the teacher–student consistency framework. Evaluation sets: VerSe 2019 validation split (40 cases) for model selection and VerSe 2019 test split (40 cases) for final reporting.
2. Fixed evaluation protocol. FSL trains only on the 80 labeled VerSe 2019 cases and evaluates on VerSe 2019 validation/test. SSL trains on the same 80 labeled VerSe 2019 cases while additionally incorporating 113 VerSe 2020 cases as unlabeled data; validation and testing remain identical to FSL (both on VerSe 2019).
3. Fairness statement. This protocol isolates the effect of unlabeled data: the only difference between FSL and SSL is whether unlabeled VerSe 2020 volumes are included during training, while evaluation sets and post-processing remain unchanged. Table 2 reports the sample counts under both protocols; for SSL, the “Training” entry is shown as “labeled + unlabeled”.

#### 2.7.2. Comparative Studies

We compare the proposed method with several representative approaches for vertebral segmentation and identification:

We additionally benchmark against established SSL baselines adapted to 3D medical segmentation, including Mean Teacher [18], UA-MT [20], CPS [27], and a UniMatch-style strong/weak consistency baseline [28]. All SSL baselines use the same labeled/unlabeled splits, backbone capacity, and post-processing to ensure a fair comparison.

1. Payer et al. [6]: a three-stage pipeline of coarse localization → identification → segmentation.
2. Sekuboyina et al. [7]: segmentation combined with location-based numbering using an improved indexing strategy.
3. nnU-Net baseline [29]: an auto-configuring segmentation framework. Since it only outputs a segmentation mask, we assign vertebral IDs post hoc using a 3D connected-component analysis in superior-to-inferior order to compute the identification accuracy.
4. SpineCLUE [17]: a recent model leveraging contrastive learning and uncertainty estimation for vertebral identification (we implement the end-to-end model following the authors’ public description).

All methods are trained and evaluated on the same VerSe 2019 labeled training and test splits. Note that some original papers used different training configurations; for fairness, we restrict all methods to use only the labeled data provided by VerSe 2019, without any extra data or pretraining. To ensure fair comparison, we applied the same connected-component instance labeling and sequential indexing (described in Section 2.6) to all segmentation outputs that lacked ID predictions (e.g., nnU-Net). In particular, the nnU-Net baseline (which produces only a binary segmentation) was followed by the same vertebra instance extraction and superior-to-inferior ID assignment procedure as our method.

#### 2.7.3. Ablation Studies

To assess the marginal contribution of each component, we design several ablation studies corresponding to the main modules and hyperparameters used in our framework. Unless otherwise specified, all ablation experiments follow the data splits described in Section 2.7.1; network architecture, optimization settings, and data augmentations are kept fixed, and only the component under investigation is changed.

1. Module add-on ablation.

We first evaluate the effect of progressively adding the identification branch and the semi-supervised consistency loss. Three configurations are compared on the VerSe 2019 validation set to quantify the effect of progressively adding the identification branch and semi-supervised learning: (i) baseline, a single-task model with only the segmentation branch trained fully supervised on labeled VerSe 2019 data; (ii) +ID, the dual-branch model that adds the identification head but is still trained purely supervised on labeled VerSe 2019 data with the consistency loss disabled; and (iii) +SSL, the full semi-supervised model that keeps the dual-branch architecture of +ID and additionally enables the teacher–student consistency training on unlabeled VerSe 2020 scans with a linearly ramped consistency weight (Section 2.4.3).

2. Pseudo-label threshold sensitivity.

With the +SSL configuration fixed, we vary the teacher pseudo-label confidence threshold τ∈{0.5,0.6,0.7,0.8,0.9,0.95} while keeping all other training details identical. We compare validation metrics as a function of τ to identify a robust operating range and a recommended threshold.

3. Mamba modules at multiple encoder layers.

To investigate where long-range modeling is most effective, we compare two placements of the Mamba module under the +SSL setting: (i) a single Mamba block inserted only at the bottleneck between an encoder and decoders (our default choice) and (ii) Mamba blocks [24] inserted at all encoder stages (“Mamba@All Encoders”). Both variants are trained with the same semi-supervised protocol. This ablation evaluates whether distributing Mamba across multiple scales yields measurable performance gains relative to the added computational cost.

4. Effect of 3D-CBAM placement.

We further examine the impact of the 3D-CBAM attention modules and where they should be placed. Under the +SSL setting, we compare three configurations: (i) no CBAM in either branch, (ii) CBAM in the ID branch only (our default design), and (iii) CBAM in both the segmentation and ID branches. All other components, including Mamba placement and consistency training, are kept unchanged. This ablation tests whether attention is primarily beneficial for the identification task, which requires discriminative features across vertebrae, or whether it also improves the segmentation branch that focuses on precise boundary delineation.

5. Replacing Mamba with a Transformer block.

Finally, to validate the choice of Mamba for long-range dependency modeling, we compare our default +SSL model (with a Mamba module at the bottleneck) against a variant in which the Mamba block is replaced by a standard 3D self-attention (Transformer [30]) block inserted at the same location. The two variants share the same dual-branch architecture and semi-supervised training setup. This ablation examines whether Mamba can match the performance of a conventional Transformer while offering lower computational complexity.

#### 2.7.4. Evaluation Metrics

Segmentation metric. The segmentation accuracy is evaluated using the mean Dice similarity coefficient (Dice), defined as follows:

(14) $$Dice=2\frac{\left|S\cap GT\right|}{\left|S\right|+\left|GT\right|}$$

where S and GT denote the predicted and ground-truth foreground voxel sets, respectively.

Identification metric. The identification accuracy is measured as instance-level accuracy (Acc), i.e., the proportion of correctly identified vertebrae over all vertebrae in the test set. Let the ground-truth index be $y\left(C\right)$ and the predicted index be $ŷ\left(Ĉ\right)$. We obtain the ground-truth instance set $C=\left\{C^{1},\;\ldots ,\;C^{m}\right\}$ by applying 3D connected-component labeling to the ground-truth vertebra mask and obtain the predicted instance set $Ĉ=\left\{{Ĉ}^{1},\;\ldots ,\;{Ĉ}^{n}\right\}$ by applying 3D connected-component labeling to the predicted binary segmentation (thresholded at 0.5), with small isolated fragments removed. The instance-wise identification accuracy ${Acc}_{ID}$ is defined in Equation (15):

(15) $${Acc}_{ID}=\frac{1}{m}\sum_{C\in C}1\left\{\exists Ĉ\in \hat{C}:\left(C,\;Ĉ\right)\in M\wedge ŷ\left(Ĉ\right)=y\left(C\right)\right\}$$

A ground-truth vertebra counts as correct only if it is matched to a predicted connected component and that component’s predicted index is correct; unmatched (missed) instances or mismatched indices are counted as errors. The matching set M is defined by an IoU criterion (Equation (16)), where τ is the mean Intersection-over-Union (IoU) matching threshold:

(16) $$M=\arg\max\sum_{\left\{\left(C,Ĉ\right)\in M\right\}}IoU\left(C,\;\hat{C}\right),\;s.t.IoU\left(C,\;\hat{C}\right)\geq \tau_{IoU}$$

Here, $1\left\{\cdot \right\}$ denotes the indicator function, and $IoU\left(C,\hat{C}\right)$ is computed between the binary masks of the individual instances C and $\hat{C}$ as the ratio of intersection to union (Equation (17)):

(17) $$IoU=\frac{\left|\hat{C}\cap C\right|}{\left|\hat{C}\cup C\right|}$$

## 3. Results

### 3.1. Quantitative Comparison

Table 3 reports the quantitative results on the test set. Overall, the fully supervised baseline (i.e., our dual-branch model without semi-supervision), trained only on VerSe 2019, achieves 89.8% Dice and 92.3% identification accuracy. After introducing unlabeled VerSe 2020 data and adopting semi-supervised training (semi-supervised), the three metrics improve to 91.6%/97.5%, i.e., gains of +1.8/+5.2 percentage points over the fully supervised baseline, demonstrating the clear benefit of leveraging unlabeled samples. Compared with representative methods, our semi-supervised model attains the best Dice and identification accuracy, outperforming Payer et al. [6] (89.8%/94.3%), SpineCLUE [17] (90.5%/97.0%), Sekuboyina et al. [7] (90.8%/95.1%), and nnU-Net [29] (91.2%/96.6%). In summary, our method substantially improves joint segmentation and identification performance without additional annotation cost, achieving the highest identification accuracy (97.5%) among all methods. The nnU-Net is a highly optimized segmentation-only framework with its own official auto-configuration. To isolate the effect of unlabeled data, our fully supervised baseline is kept identical to the SSL setting (see Discussion). Specifically, our supervised baseline and all SSL variants use the same preprocessing (resampling to 1.0 mm, HU clipping to [−500,1500], and [0,1] normalization), the same patching strategy (128 × 64 × 64 with identical padding/cropping), and the same optimization schedule (Section 2.6). By contrast, the nnU-Net benchmark follows the official nnU-Net auto-configuration, which may select different patch sizes, augmentation policies, and optimization hyperparameters to maximize the segmentation Dice. Therefore, the reported SSL gain (+1.8 Dice) should be interpreted relative to our supervised baseline under identical settings, whereas the remaining absolute gap to nnU-Net mainly reflects architectural and training/auto-configuration differences (see Section 4).

We additionally benchmark against established SSL baselines adapted to our 3D setting under the same labeled/unlabeled split (VerSe 2019 labeled + VerSe 2020 unlabeled), backbone capacity, and inference post-processing. Mean Teacher [18], UA-MT [20], CPS [27], and a UniMatch-style weak/strong consistency baseline [28] achieve 90.9%/96.0%, 91.1%/96.4%, 91.3%/96.7%, and 91.4%/96.9% (Dice/accuracy), respectively. Our method consistently outperforms all these SSL baselines, improving the strongest SSL baseline (UniMatch-style) by +0.2 Dice and +0.6 accuracy, while retaining the highest identification accuracy (97.5%) among all compared methods. In summary, our approach substantially improves joint segmentation and identification performance without additional annotation cost.

### 3.2. Ablation Studies

#### 3.2.1. Effect of Main Modules

**Figure 2:**
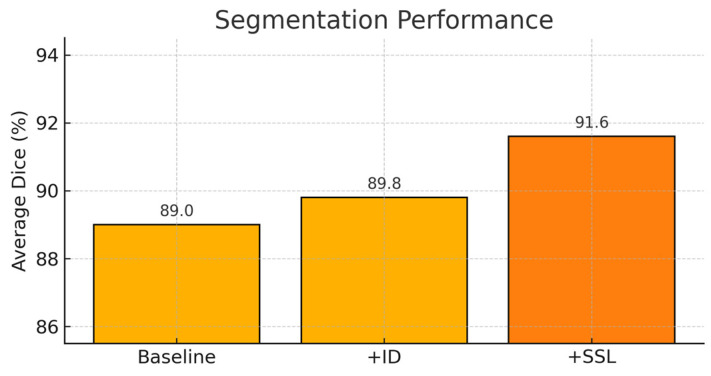
Ablation study showing performance gains from the main modules. Average Dice on the validation set for three configurations: baseline (segmentation-only, supervised), +ID (dual-branch segmentation + identification, supervised), and +SSL (dual-branch model with additional semi-supervised consistency on unlabeled data). Both adding the ID branch and further enabling SSL lead to progressive improvements, with the largest gain obtained when SSL is applied on top of the multi-task architecture.

Figure 2 shows that each step yields a clear improvement. Starting from the segmentation-only baseline, adding the identification branch (+ID) improves the mean Dice from 89.0% to 89.8% (≈+0.8 points) and increases the identification accuracy from 92.3% to about 94.5%. This indicates that joint optimization with the ID branch brings a modest but consistent gain for segmentation rather than harming it. The additional branch encourages the network to better distinguish adjacent vertebrae and acts as a form of multi-task regularization, reducing overfitting and diminishing “sticking” errors between neighboring vertebrae.

Building on this multi-task architecture, introducing semi-supervised consistency (+SSL) further boosts the mean Dice to 91.6% (+1.8 points over +ID, +2.6 points over the baseline) and raises the identification accuracy to 97.5%. In this setting, weak/strong view consistency and high-confidence pseudo-labels from the unlabeled CTs provide an extra source of supervision, making the model more robust to contrast variations and noise. Taken together, the ablation demonstrates two distinct contributions: (i) the multi-task design (+ID) already brings a measurable improvement over a segmentation-only baseline, and (ii) adding SSL on top of this design (+SSL) yields a substantially larger gain by exploiting the unlabeled data.

#### 3.2.2. Effect of Pseudo-Label Confidence Threshold

**Figure 3:**
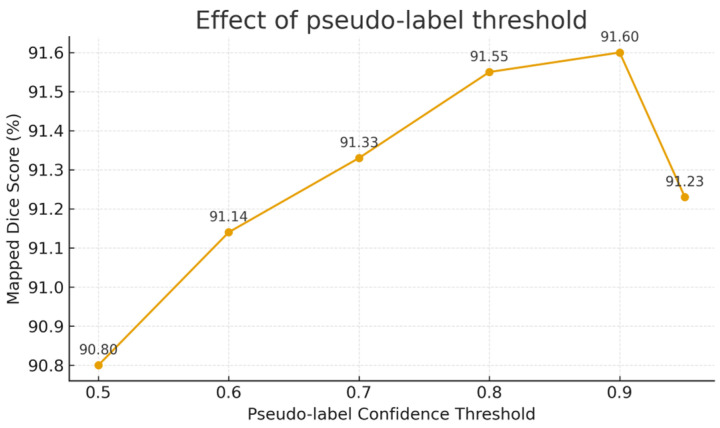
Impact of the pseudo-label confidence threshold.

For the pseudo-label confidence threshold, Figure 3 plots the segmentation Dice as the teacher confidence threshold τ varies from 0.5 to 0.9. Thresholds that are too low (<0.6) admit many low-quality pseudo-labels, degrading performance; as τ approaches ~0.9, Dice peaks at ≈91.6%. When τ is raised to 0.95, the number of usable pseudo-labels drops, and the performance slightly declines (91.23%). We, therefore, choose τ=0.9 as a trade-off, balancing pseudo-label quality and quantity. Moreover, because we employ uncertainty-based masking, the strong-view pseudo-label regions that actually contribute to the loss are more precise; even with τ=0.9, the model mainly leverages high-confidence pseudo-labels, further improving the training efficacy. Overall, the ablations verify that multi-task learning, contrastive-style pretraining, and semi-supervision all contribute to better vertebra segmentation and identification, with semi-supervision providing the largest gain and multi-task learning the next. These strategies are complementary, enabling strong performance under limited annotations.

#### 3.2.3. Mamba Modules at Multiple Encoder Layers

**Figure 4:**
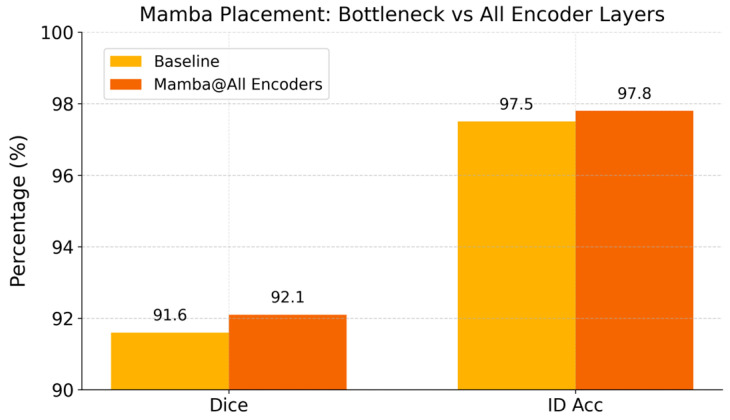
Performance with Mamba modules inserted at all encoder stages vs. only at the bottleneck.

For Mamba placement, Figure 4 compares using Mamba modules at all encoder levels vs. only at the bottleneck. Inserting a Mamba at every encoder stage (“Mamba@All Encoders”) yields slightly higher Dice (92.1% vs. 91.6%) and ID accuracy (97.8% vs. 97.5%) than using a single bottleneck Mamba. This suggests that modeling a long-range context at multiple scales can provide marginal gains. However, the improvement is modest, indicating that a single well-placed Mamba at the bottleneck already captures most of the global dependency information. Given the increased computational cost of inserting Mamba at every level, our default choice of one bottleneck Mamba offers a favorable trade-off between complexity and performance.

#### 3.2.4. Effect of 3D-CBAM Placement

We conducted an ablation experiment to verify the decision to apply 3D-CBAM only in the identification branch. Three configurations were compared under the semi-supervised (SSL) setting: (i) no CBAM in either branch, (ii) CBAM in the ID branch only (our default design), and (iii) CBAM in both the segmentation and ID branches. Table 4 summarizes the validation results. We observe that adding CBAM to the identification branch yields a notable improvement in the identification accuracy (+1–2%) with minimal effect on segmentation performance, whereas further applying CBAM to the segmentation branch offers no additional gain. In fact, the “CBAM in both” setting shows essentially no ID improvement and a negligible Dice change (<0.1% drop, e.g., 91.6% to 91.5%) compared to using CBAM only in the ID branch. This suggests that channel/spatial attention is most beneficial for the identification task, which relies on learning discriminative features across vertebrae, whereas the segmentation task—focused on precise boundary delineation—does not appreciably benefit from CBAM and may even be negatively affected. These results support our design choice to deploy 3D-CBAM in the identification decoder only.

#### 3.2.5. Replacing Mamba with a Transformer

**Figure 5:**
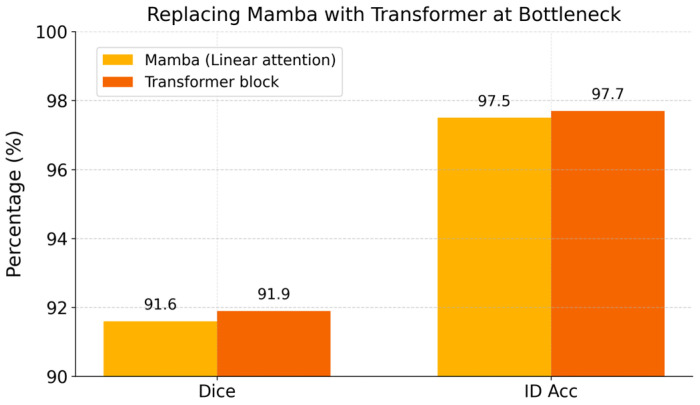
Ablation replacing the Mamba module with a Transformer block at the bottleneck.

The Figure 5 bar chart compares the proposed model (with the efficient Mamba module for linear-complexity attention) against a variant using a standard Transformer self-attention module in the same location. The two models achieve very similar performance: the Transformer variant attains Dice of ≈91.9% and ID accuracy of ≈97.7% compared to 91.6% and 97.5% with Mamba. These differences (~+0.3 in Dice and +0.2% in accuracy) are minimal, indicating that the global self-attention mechanism yields no significant advantage over Mamba in this task. Given Mamba’s comparable accuracy but lower computational overhead (linear vs. quadratic time), it is the more efficient choice for capturing long-range context in our framework. In summary, the Mamba module provides nearly the same benefit as a full Transformer block for vertebra context modeling at a fraction of the cost.

### 3.3. Cross-Domain Generalization

To examine the model’s cross-domain robustness, we evaluated the trained VerSe model on the CTSpine1K [29] dataset (an external multi-source dataset) without any additional fine-tuning. As summarized in Table 5, the overall performance decreases from 91.6% Dice/97.5% identification accuracy on the VerSe test to 87.4% Dice/90.2% accuracy on CTSpine1K, indicating a moderate domain-shift effect (scanner protocols, reconstruction kernels, and patient populations).

We further report results stratified by anatomical region (cervical/thoracic/lumbar) to characterize where cross-domain errors concentrate (Table 5). On CTSpine1K, the cervical region exhibits the largest drop (e.g., 86.0% Dice/86.5% accuracy), which is consistent with frequent partial FOV truncation (e.g., scans starting at C3–C4) and the resulting global numbering offsets. The thoracic region shows intermediate performance (e.g., 87.2% Dice/90.5% accuracy), where errors more often manifest as adjacent-vertebra confusion in morphologically similar mid-thoracic levels. The lumbar region is relatively more stable (e.g., 89.8% Dice/94.5% accuracy), but we observed occasional failure cases associated with severe degenerative changes (osteophytes, collapsed discs) and transitional vertebrae (e.g., S1) that can introduce local ID inconsistencies or label-mapping ambiguity. These observations suggest that cross-domain degradation is not uniform: errors concentrate at regions more prone to truncation, ambiguous morphology, or anatomical variants.

Qualitative examples are provided in the revised figures, illustrating typical failure modes: (i) partial FOV truncation causing missing superior vertebrae and a downstream ID shift; (ii) severe degenerative changes leading to fused/fragmented components and localized mis-numbering; and (iii) transitional vertebrae (e.g., an extra lumbar vertebra) causing systematic off-by-one labeling under the standard C1–L5 reporting protocol.

## 4. Discussion

This study demonstrates the effectiveness of semi-supervised learning for vertebral segmentation and identification in spinal CT. Compared with prior pipelines, our end-to-end multi-task model fully integrates segmentation and numbering, avoiding the error amplification typical of staged methods. On the VerSe benchmarks, our approach surpasses the best reported results in both Dice and identification accuracy. However, cross-site evaluation revealed that performance can degrade under distribution shifts (Section 3.3), underscoring the need for domain adaptation.

Cross-domain analysis notes the following: for both VerSe and CTSpine1K, we use the same intensity preprocessing (HU clipping to [−500, 1500] and [0, 1] normalization after resampling) and the same evaluation protocol. The cervical/thoracic/lumbar (C/T/L) breakdown is computed using identical vertebral-level criteria across datasets. Therefore, the observed domain shift mainly stems from differences in scanner/protocol, field-of-view truncation, and anatomical distribution rather than from inconsistent normalization.

Nonetheless, several limitations remain. Firstly, our method still requires a certain amount of labeled data for initialization. Secondly, we used only the unlabeled data provided by VerSe; future work could incorporate larger, more diverse unlabeled spinal CT corpora to further exploit SSL. Thirdly, generalization across scanners and acquisition protocols may be affected by domain shifts; domain adaptation techniques could alleviate this.

We acknowledge that our fully supervised baseline segmentation Dice is slightly lower than the nnU-Net [29] benchmark. Importantly, our supervised baseline is designed as a matched control for the SSL study: it uses identical preprocessing, patching (128 × 64 × 64), and the same optimization schedule as the SSL setting, so that the reported SSL gain (+1.8 Dice) reflects the effect of adding unlabeled data rather than changes in training configuration. By contrast, nnU-Net is a segmentation-only framework that automatically selects task-specific hyperparameters (e.g., patch size, augmentation strength, and optimizer/schedule) to maximize Dice. In addition, our architecture is multi-task (segmentation + identification) with a dual-branch decoder and loss balancing; this can slightly reduce the effective capacity/optimization focus for pure segmentation compared with a single-task nnU-Net model. We have added this clarification to contextualize the baseline positioning and to make the comparison fair and interpretable.

In future work, we plan to (1) incorporate richer anatomical priors (e.g., plausible spine length ranges and typical vertebra counts) to improve robustness under congenital variants or partial scans; (2) scale up semi-supervised training with larger unlabeled datasets to mine more latent information; and (3) study domain adaptation across hospitals, devices, and imaging parameters to enhance real-world generalization. Overall, our results highlight the substantial potential of semi-supervised deep learning in medical imaging, with a promise for more reliable clinical decision support.

## 5. Conclusions

We address the challenging task of automatic vertebral segmentation and identification in CT with a semi-supervised approach that (1) unifies voxel-wise segmentation and per-vertebra identification within a single 3D multi-task network, enabling truly end-to-end prediction, and (2) integrates weak/strong consistency and pseudo-label filtering to make effective use of unlabeled data, improving generalization under limited annotations. Experiments on the public VerSe 2019 and 2020 benchmarks validate the superiority of our method: both segmentation accuracy and identification accuracy reach or exceed state-of-the-art levels. The proposed framework can help clinicians rapidly obtain 3D vertebral masks with consistent IDs, with potential applications in surgical planning and postoperative assessment.

## Figures and Tables

**Table 1:** Number of scans and vertebrae in the VerSe dataset.

	Dataset	Training	Validation	Test	Total
Number of Scan	VerSe 2019	80	40	40	160
	VerSe 2020	113	103	103	319
Number of Vertebrae	VerSe 2019	220	884	621	1725
	VerSe 2020	581	2255	1305	4141

**Table 2:** Supervised and semi-supervised setups based on VerSe 2019/2020 (number of cases).

Split	FSL (VerSe 2019 only)	SSL (VerSe 2019 + 2020)
Training	80 (80 labeled-from 2019)	193 (80 ^1^ labeled-from 2019, 113 unlabeled-from 2020)
Validation	40 (40 labeled-from 2019)	40 (40 labeled-from 2019)
Test	40 (40 labeled-from 2019)	40 (40 labeled-from 2019)
Total	160 (160 labeled-from 2019)	273 (160 labeled-from 2019, 113 unlabeled-from 2020)

^1^ For ablation experiments using a specific labeled proportion (e.g., 0.75), the supervised branch uses 60 labeled cases from the VerSe 2019 training split, while the remaining 20 cases, together with the 113 VerSe 2020 cases, are treated as unlabeled; the validation and test sets remain unchanged.

**Table 3:** Quantitative performance comparison on VerSe datasets.

Method	Dataset	Dice (%)	Accuracy (%)
Payer et al. [6]	2019 labeled	89.8	94.3
Sekuboyina et al. [7]	2019 labeled	90.8	95.1
nnU-Net [29]	2019 labeled	91.2	96.6
SpineCLUE [17]	2019 labeled	90.5	97.0
Proposed (FSL)	2019 labeled	89.8	92.3
Mean Teacher (SSL) [18]	2019 labeled + 2020 unlabeled	90.9	96.0
UA-MT (SSL) [20]	2019 labeled + 2020 unlabeled	91.1	96.4
CPS (SSL) [27]	2019 labeled + 2020 unlabeled	91.3	96.7
UniMatch-style (SSL) [28]	2019 labeled + 2020 unlabeled	91.4	96.9
Proposed (SSL)	2019 labeled + 2020 unlabeled	91.6	97.5

**Table 4:** Ablation results for 3D-CBAM placement. All models are trained under the semi-supervised (SSL) configuration. Applying CBAM in the ID branch improves the identification accuracy, while further adding it to the segmentation branch offers no significant benefit.

CBAM Placement	Dice (%)	Accuracy (%)
No CBAM	91.2	96.4
CBAM in ID branch only	91.6	97.5
CBAM in both branches	91.5	97.4

**Table 5:** Cross-domain evaluation stratified by anatomical region. Performance of the VerSe-trained model on the CTSpine1K dataset (unseen during training). For reference, results on the VerSe test set are also shown. A moderate drop in accuracy is observed on CTSpine1K due to domain shift.

Model (Training → Testing)	Region	Dice (%)	Accuracy (%)
Proposed (VerSe → VerSe test)	Overall	91.6	97.5
	Cervical (C1–C7)	91.0	97.0
	Thoracic (T1–T12)	91.5	97.6
	Lumbar (L1–L5)	92.8	98.2
Proposed (VerSe → CTSpine1K test)	Overall	87.4	90.2
	Cervical (C1–C7)	86.0	86.5
	Thoracic (T1–T12)	87.2	90.5
	Lumbar (L1–L5)	89.8	94.5

Region-wise metrics are computed at the vertebra-instance level by grouping ground-truth instances into cervical (C1–C7), thoracic (T1–T12), and lumbar (L1–L5). When transitional vertebrae are present (e.g., S1), we follow the same reporting protocol as VerSe (C1–L5) and explicitly apply a consistent label-mapping rule.

## Data Availability

The VerSe’19 and VerSe’20 datasets analyzed in this study are available for download at https://osf.io/nqjyw/ (accessed on 11 November 2025) and https://osf.io/t98fz/ (accessed on 11 November 2025), respectively (see https://github.com/anjany/verse for details (accessed on 11 November 2025)). The CTSpine1K dataset analyzed in this study is available for download at https://huggingface.co/datasets/alexanderdann/CTSpine1K/tree/main (accessed on 11 November 2025) (see https://github.com/MIRACLE-Center/CTSpine1K (accessed on 11 November 2025) for details). To support reproducibility, we have publicly released the core implementation at https://github.com/linbi2333/SSVS (accessed on 11 November 2025).

## Abbreviations

The following abbreviations are used in this manuscript:

CT	Computed tomography
SSL	Semi-supervised learning
FSL	Fully supervised learning
CBAM	Convolutional block attention module
EMA	Exponential moving average

## Appendix A

### Appendix A.1

**Table A1:** Notation and symbols used in the loss definitions.

Symbol	Meaning
$L_{toal}(t)$	Total training objective at epoch t.
$L_{sup}$	Supervised loss on labeled data.
$L_{cons}$	Self-supervised consistency loss on unlabeled data.
$L_{cons}^{seg}$	Segmentation consistency loss on unlabeled data.
$L_{cons}^{cls}$	Identification consistency loss on unlabeled data.
$L_{seg}$	Segmentation loss (deep supervision across scales).
$L_{cls}^{KL}$	Identification distribution alignment loss (KL divergence between target and predicted heatmap distributions).
$L_{cls}^{inst}$	Identification instance-consistency loss (mean Dice over instances after splitting).
$\alpha_{cls}$	Weight of identification loss in the supervised objective.
$\alpha_{seg}$	Internal weighting factor between Dice and CE inside the segmentation loss.
$w_{s}$	Scale weight for deep supervision at resolution scale s (Σ_{s∈S} w_s = 1).
$\Omega_{s}$	Voxel grid at resolution scale s.
x	Voxel index (position) in a voxel grid.
$g_{s}\left(x\right)$	Ground-truth foreground label at voxel x (0 background, 1 vertebra).
$p_{s}\left(x\right)$	Predicted foreground probability at voxel x.
ε	Small constant to avoid division by zero (ε = 10^−6).
$\lambda \left(t\right)$	Epoch-dependent weight for consistency training (ramp-up schedule).
$\lambda_{cons}$	Maximum consistency weight (λ_cons = 0.5).
$E_{cons}$	Epoch when consistency training starts (warm-up length; E_cons = 40).
$E_{ramp}$	Epoch when ramp-up reaches its maximum (E_ramp = 80).
$\tau_{cls}$	Confidence threshold for ID pseudo-label filtering (ramped 0.30 → 0.55).
$\tau_{seg}$	Confidence threshold for segmentation pseudo-label filtering (ramped 0.50 → 0.90).
T	Temperature used in teacher heatmap softmax for ID pseudo-labels (T = 0.5).
$\theta_{student}$	Student network parameters.
$\theta_{ema}$	EMA teacher network parameters.
α	EMA decay factor in teacher update (α = 0.99).
$\sigma \left(\cdot \right)$	Sigmoid function used to map segmentation logits to probabilities.
K	Number of vertebra classes (e.g., C1–L5) used for identification.
$w_{k}$	Class-frequency weight for class k in identification consistency loss.
$\Omega_{seg}$	Set of high-confidence voxels used for segmentation consistency.
$\Omega_{cls}^{k}$	Set of high-confidence voxels used for identification consistency for class k.
${ŷ}_{x}^{T}$	Teacher binary pseudo-label at voxel x for segmentation consistency.
$y_{x}^{T}$	Teacher pseudo-class label at voxel x for identification consistency.
$y_{x}^{S}$	Student class-probability vector at voxel x for identification consistency.
$z_{x}^{S}$	Student segmentation logit at voxel x.

### Appendix A.2

**Table A2:** Summary of weak versus strong augmentations (parameter ranges).

Category	Operation	Probability (p)	Magnitude/Parameters
Geometric	Flip (X/Y axis)	0.5	-
	Flip (Z axis)	0.25	-
	Rotation (XY plane)	1.0	$\theta \in [-22.5^{{}^{\circ}},+22.5^{{}^{\circ}}]$
Intensity	Scale & Shift	0.5	$\alpha \in \left[0.8,1.2\right],\beta \in [-.1,0.1]$
	γ-Correction	0.5	γ in [0.7, 1.5]
	CLAHE (Slice-wise)	0.5	Clip limit = 0.03
	Unsharp Masking	0.5	Amount ∈ [0.5, 1.5], $\sigma_{blur}=1.0$
Noise & Blur	Gaussian Noise	0.5	σ∈[0,0.025]
	Speckle Noise	0.3	σ∈[0,0.05]
	Salt-and-Pepper	0.3	σ∈[0.001,0.01]
	Poisson Noise	0.3	-
	Gaussian Blur	0.5	σ∈[0.75,1.25]
	Median Filter	0.5	Kernel size = 3
